# Extraabdominal fibromatosis in retroperitoneal space

**DOI:** 10.1186/1477-7819-2-33

**Published:** 2004-10-03

**Authors:** Akira Kikkawa, Akira Kido, Tsukasa Kumai, Toru Hoshida

**Affiliations:** 1 Department of Urology, Hannna Central Hospital, Nara, Japan; 2 Divison of Orthopedics and Traumatology, Medical Center for Emergency and Critical Care, Nara Prefectural Nara Hospital, Nara, Japan; 3 Department of Orthopedic Surgery, Nara Medical University, Nara, Japan

## Abstract

**Background:**

Fibromatosis or desmoid tumor covers a broad spectrum of benign fibrous tissue proliferations. It is characterized by infiltrative growth and a tendency towards recurrence; however, unlike sarcoma, it never metastasizes.

**Case presentation:**

We report on a case of extraabdominal fibromatosis originating from the retroperitoneal space in a 43-year-old woman. Seven years earlier she had undergone ureterolysis and ureteroureterostomy for ureteral obstruction. Computed tomography revealed a tumor between the iliocostalis and the psoas muscle. Histopathological evaluation revealed uniform proliferation of spindle cells, with a moderate amount of collagen fibers, suggesting extraabdominal fibromatosis (desmoid tumor). The tumor was surgically resected, and since then, the patient has remained asymptomatic without any restrictions of daily living activities and without any signs of tumor recurrence during the two-year follow-up.

**Conclusions:**

Complete resection is the treatment of choice. Adjuvant therapy using non steroidal anti-inflammatory agents, tamoxifen, interferon, anti-neoplastic agents, and radiotherapy, either alone or in combination finds application for unresectable or recurrent cases.

## Background

The term "fibromatosis" covers a broad spectrum of benign fibrous tissue proliferations, the biological behavior of which is similar to both benign fibrous lesions and fibrosarcoma. Like fibrosarcoma, fibromatosis is characterized by infiltrative growth and a tendency towards recurrence; however, unlike sarcoma, it never develops metastasis [1]. Therefore, the most important strategy is to prevent direct invasion into adjacent tissues. Extraabdominal fibromatosis principally originates from the connective tissue of muscles and the overlying fascia or aponeurosis. It may occur in a variety of anatomical locations, including the muscles of the shoulder, the chest wall and back, thigh, and head and neck. However, solitary occurrence is rare in retroperitoneal space [1,2]. Here, we report on a case of extraabdominal fibromatosis in the retroperitoneum. Resection was successfully performed, and the patient has been tumor-free for two years after surgery.

## Case presentation

**Figure 1:**
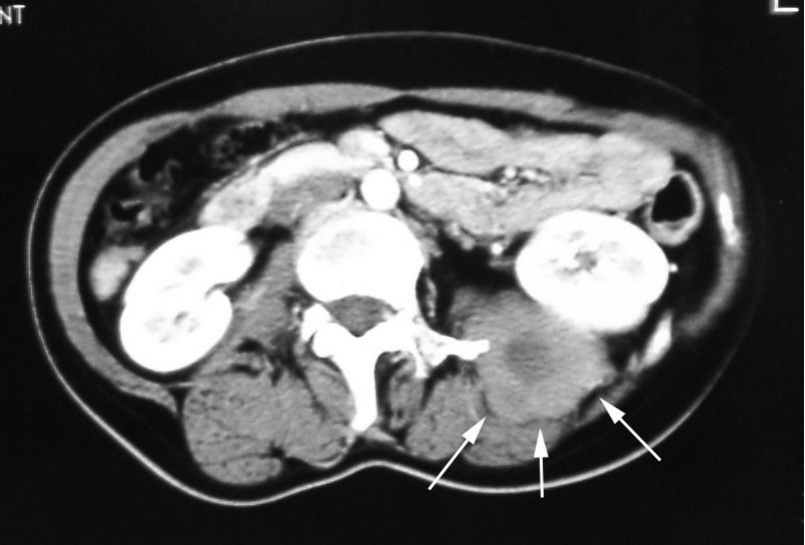
Contrast enhanced computed tomography showing the tumor location between iliocostalis and psoas muscles in retroperitoneal space.

**Figure 2:**
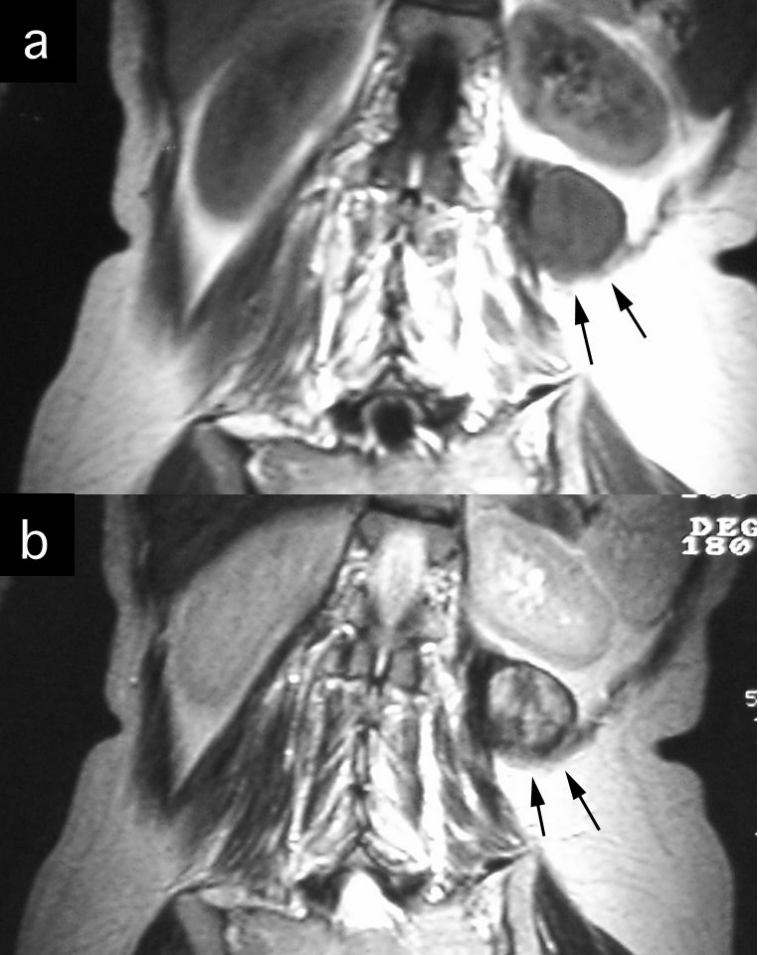
Coronal magnetic resonance imaging demonstrating the tumor beneath the left kidney.

**Figure 3:**
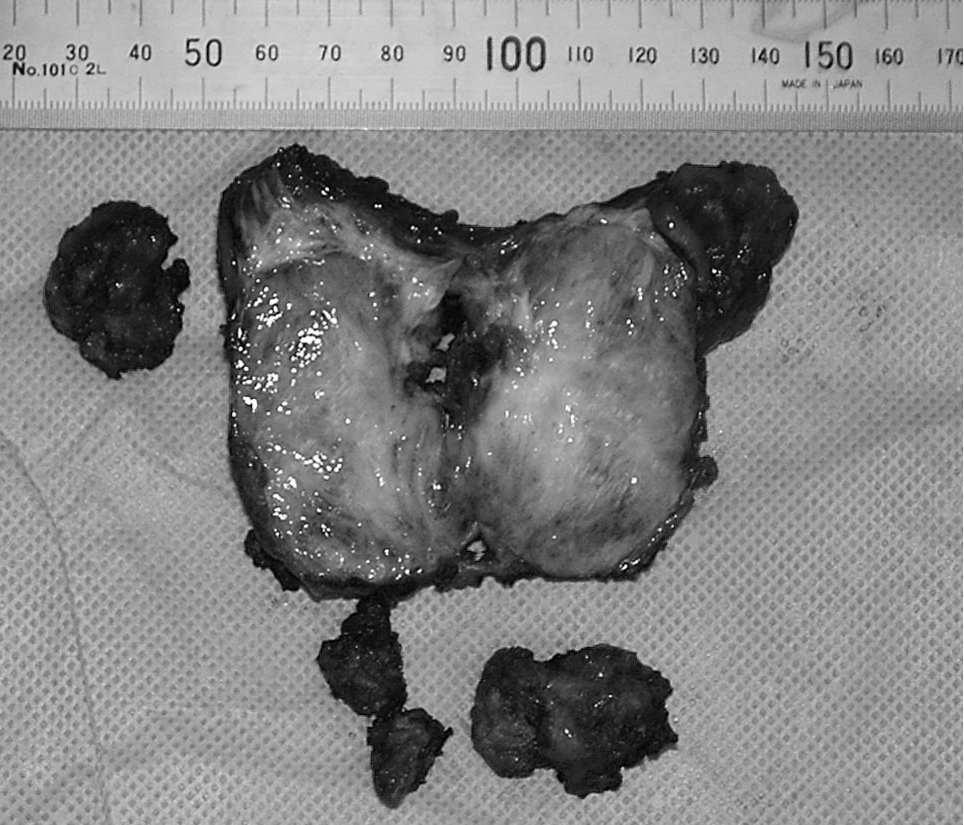
Gross appearance of the resceted tumor. The cut surface homogeneously appears gray and glossy.

**Figure 4:**
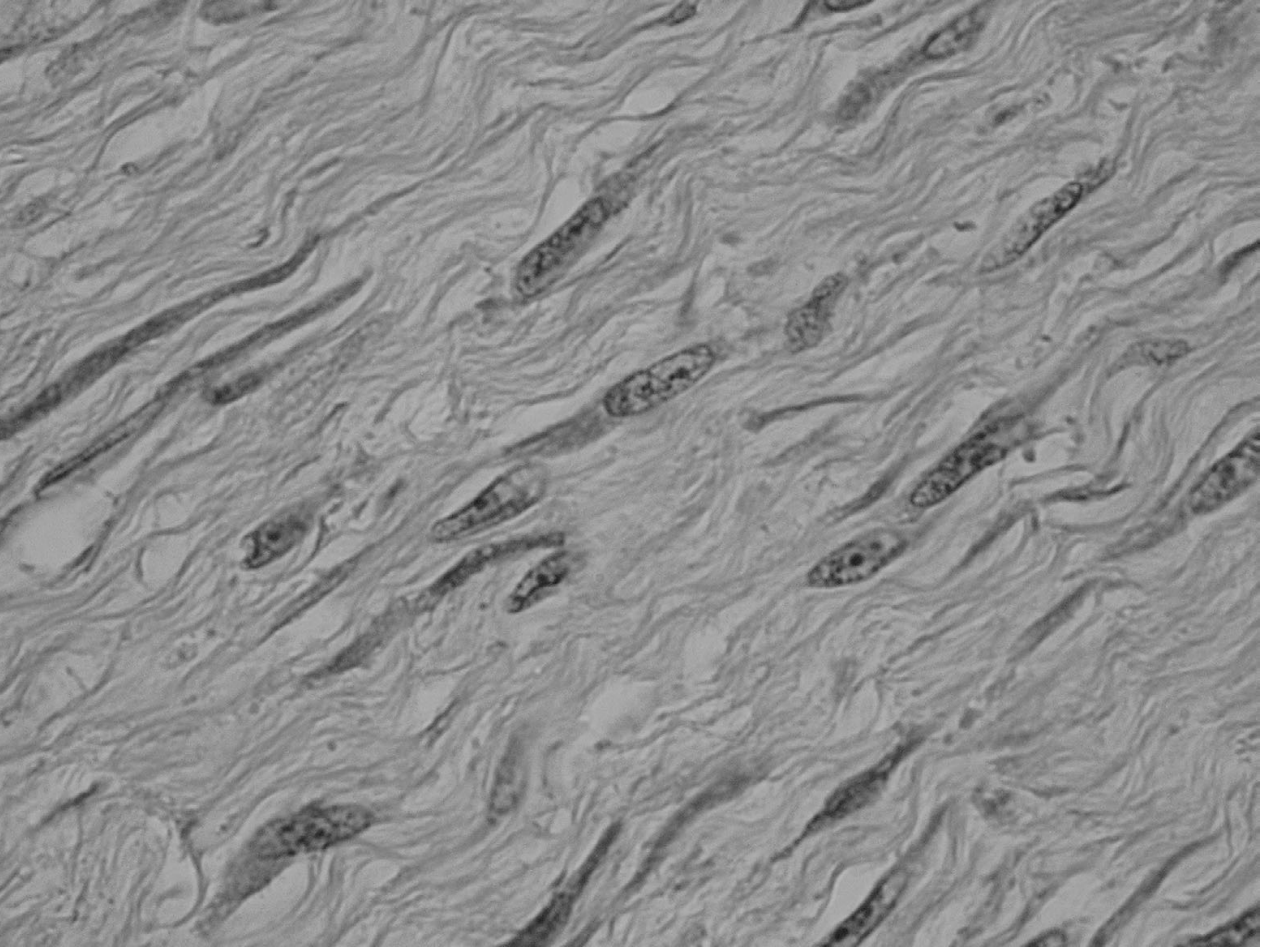
Photomicrograph showing the tumor composed of uniform spindled proliferation with moderate account of collagen fibers (hematoxylin and eosin ×400).

A 43-year-old woman with a history of schizophrenia since 1982, and a history of hospitalization to help the patient to acquire social communication abilities, at the age of 23 presented with slight pain on her left flank and back. In 1995, she was treated with ureterolysis and ureteroureterostomy because of left-sided ureteral obstruction. Histological evaluation of the biopsy revealed benign fibrous tissue proliferations; however, no further evaluation and surgical excision was planned as her mental state was deteriorating. She was put on regular follow-up with computed tomography (CT) scans. In May 2002, she was referred from the psychiatric hospital to our Department of Urology, as the tumor tended to grow. CT scan with contrast enhancement revealed a tumor located between iliocostalis and psoas muscles in retroperitoneal space. The peripheral part of the tumor was enhanced, while the central part did not. The left paravertebral muscles around the tumor showed atrophy. The medial margin of the tumor was deformed by a left transverse process of the second lumber spine, suggesting invasive behavior (Figure 1). Coronal magnetic resonance imaging (MRI) demonstrated the tumor to be located beneath the left kidney. The central part of the tumor was found to be of iso/low intensity in the T1-weighted phase and of heterogeneously high intensity in the T2-weighted phase. The marginal part showed very low intensity in both phases (Figure 2). In May 2002, needle biopsy was performed and revealed that the tumor consisted of well-proliferated spindle cells rich in collagen fibers, an observation that was inconsistent with the histological pictures made in 1995. In June 2002, resection was performed using a paraspinal approach. Although the tumor strongly adhered to adjacent tissues, including the urinary tract and peritoneum, it was marginally resected, including paravertebral muscles and part of the spine. As the kidney was less affected by the tumor, ablation posed no problem. On gross examination, the cut surface appeared homogeneously gray and glossy (Figure 3). Histologically, a uniform proliferation of spindle cells with a moderate amount of collagen fibers led to a diagnosis of extraabdominal fibromatosis in the retroperitoneal space (Figure 4). No adjuvant treatment was given and during the two years of follow-up, the patient has remained asymptomatic, with no restrictions of daily living. There were no clues as to recurrence of the tumor in computed tomography.

## Discussion

Extraabdominal fibromatosis may occur in a variety of anatomic locations; the principle sites of the involvement are the shoulder, chest wall and back, thigh and head and neck. Origin of extraabdominal fibromatosis from any mesenchymal tissue is now well recognized [1,2]. Several authors have reported retroperitoneal fibromatosis in patients with familial adenomatous polyposis (Gardner syndrome) [2,4-6], however, solitary occurrence of fibromatosis is very rarely reported [1,2,7-9].

Our patient did not have a family history and upper gastrointestinal endoscopy, colonoscopy, or opthalmoscopy were normal suggesting that our patient may be negative for the syndrome. The exact histological origin of the tumor remains to be verified. The findings of CT suggested an origin from paravertebral muscles. Interestingly, this assumption was corroborated by a computed tomography performed in April 2000, which revealed that the previous tumor was located intramuscularly

Principally, complete resection is the therapy of choice for this type of tumors [10]. Adjuvant therapy using non steroidal anti-inflammatory drugs (NSAIDs), tamoxifen, interferon, anti-neoplastic agents, radiation, and a combinations of these, have been reported for cases that are difficult to resect [1], the exact benefit offered by them is not known due to thin literature. Radiation therapy is accepted as an effective treatment after incomplete resection [11,12]. Recently, preoperative radiotherapy was reported to be useful for the local control [13].

In our case the tumor detection was delayed because the psychiatric status of our patient which has been unstable for several years. As wide resection of the tumor reduces the risk of recurrence, an early diagnosis is required for this type of tumor, which is difficult as most of these patients are asymptomatic. While the silent area contains several vital organs, extraabdominal fibromatosis should be considered for the differential diagnosis for such a lesion.

## Competing interests

The authors declare that they have no competing interests.

## Authors' contributions

AKik and AKid performed the operation, are responsible for the clinical work and helped with the preparation of the manuscript. TK is the orthopedic consultant and helped with the preparation and editing of the manuscript. TH coordinated and drafted the manuscript. All authors read and approved the final manuscript.
